# Chemical restraint in routine clinical practice: a report from a general hospital psychiatric ward in Greece

**DOI:** 10.1186/1744-859X-10-4

**Published:** 2011-02-24

**Authors:** Nikolaos Bilanakis, Georgios Papamichael, Vaios Peritogiannis

**Affiliations:** 1 Department of Psychiatry, General Hospital of Arta, Arta, Greece; 2 Private Practice, Ioannina, Greece

## Abstract

**Background:**

There is a dearth of studies regarding chemical restraint in routine clinical psychiatric practice. There may be wide variations between different settings and countries.

**Methods:**

A retrospective study on chemical restraint was performed in the 11-bed psychiatric ward of the General Hospital of Arta, in northwestern Greece. All admissions over a 2-year-period (from March 2008 to March 2010) were examined.

**Results:**

Chemical restraint was applied in 33 cases (10.5% of total admissions). From a total of 82 injections, 22 involved a benzodiazepine and/or levomepromazine, whereas 60 injections involved an antipsychotic agent, almost exclusively haloperidol (96.7% of cases), usually in combination with a benzodiazepine (61.7% of cases). In 36.4% of cases the patient was further subjected to restraint or seclusion.

**Conclusions:**

In our unit, clinicians prefer the combined antipsychotic/benzodiazepine regimen for the management of patients' acute agitation and violent behaviour. Conventional antipsychotics are administrated almost exclusively and in a significant proportion of cases further coercive measures are applied. Studies on the practice of chemical restraint should be regularly performed in clinical settings.

## Introduction

Coercive measures are commonly used in psychiatric treatment for the management of behaviour in patients who are disturbed, although the need for alternatives has been widely recognised [1]. Most authors focus on seclusion and physical restraint, whereas chemical (pharmaceutical) restraint or rapid tranquilisation has gained little attention in the recent literature. We have previously reported on the practice of restraint and seclusion in Greece [2] and the patients and families attitudes towards coercive measures [3]. Here, we report on the practice of chemical restraint in a psychiatric ward within a general hospital.

## Methods

A total of 314 admissions in the 11-bed psychiatric ward of the General Hospital of Arta in northwestern Greece from a 2-year-period (March 2008 to March 2010) were examined retrospectively. Rapid tranquilisation (defined as emergency intramuscular drug administration for the management of patients' acute agitation and violent behaviour) was applied in 33 cases (10.5% of total admissions), involving 24 patients in different hospitalisation events. A total of 14 patients (58.3%) were male and the age range of the patients was between 25 and 75 years (mean 46.9 years). Regarding diagnosis, 16 patients (66.7%) suffered from schizophrenia and related psychoses and 4 patients (16.7%) from bipolar disorder, being in a manic episode at admission. On 21 occasions (63.6%), patients had been admitted involuntarily.

## Results

The total number of injections given was 82. In the majority of cases (24 cases, 72.7%) only 1 or 2 injections had been required for the management of agitation and violent behaviour. A total of 22 injections involved a benzodiazepine (mostly diazepam, due to limited availability of lorazepam in our unit) and/or levomepromazine, a sedative agent with only weak antipsychotic effects. In all, 60 injections (73.2%) involved an antipsychotic agent, almost exclusively haloperidol (58 injections or 96.7%), usually in combination with a benzodiazepine (in 37 out of 60 cases, 61.7%). The administrated dosages were greater than 300 mg equivalent to chlorpromazine on eight occasions.

In 12 cases (36.4%) the patient was further subjected to restraint or seclusion. Such combination of coercive measures was clinically justified for the most effective management of the patients' aggressive and violent behaviour, and is commonly used in inpatient psychiatric units throughout the world [4,5].

Statistical analysis was not performed due to the limited number of cases.

## Discussion

These results suggest that clinicians in our unit tend to use antipsychotic agents, usually in combination with a benzodiazepine, for the management of severely disturbed patients, whereas in a significant proportion of cases further coercive measures are applied. Conventional agents are almost exclusively preferred despite the availability of atypical antipsychotics parenteral formulations in our unit. The reasons for this are not known, but we assume that practicing clinicians are more familiar with the parenteral use of conventional antipsychotics since they have been using these agents for long. Moreover, they may be convinced of the efficacy and safety of these agents according to their clinical experience. However, in recent years parenteral formulations of several atypical antipsychotics have been developed, and they are now available in the market. These agents have been reported to be safe and better tolerated than conventional antipsychotics, and they are now considered as effective and safe treatments for acute agitation [6]. However, the evidence for the effectiveness of these agents has been questioned because they have been studied in patient populations that are less severely agitated than 'real world' patients [7]. The administrated doses of conventional antipsychotics were rather conservative (< 300 mg equivalent to chlorpromazine), probably due to the preferred combination with a benzodiazepine.

The results of our study may be considered clinically relevant, because they involve every day clinical practice, yet they must be viewed with caution. The number of patients is small and the setting is a psychiatric ward within a general hospital. As this is the first report on the use of rapid tranquilisation from Greece there are no relative data available for national comparisons, thus conclusions cannot be reached. The tendency for the use of conventional antipsychotics for the management of inpatient agitation may not be the case in other general hospital psychiatric wards or psychiatric hospitals in our country. It should be noted, however, that the practice of chemical restraint in our unit corresponds well to the recommendations of the two existing guidelines, the UK National Institute of Clinical Excellence (NICE) guideline on the short-term management of violent behaviour [8] and the German guideline on treatment of aggressive behaviour [9]. According to these guidelines there is good evidence that benzodiazepines (mainly lorazepam) and antipsychotics are effective and reasonably safe for use in rapid tranquilisation. The limited availability of lorazepam in our unit provides a rationale for the extensive use of antipsychotics (73.2% of injections) as emergency medications. Regarding the application of additional coercive measures to a significant proportion of patients (36.4%), it should be mentioned that it is not contraindicated according to the NICE guideline [8].

To date there are no national guidelines in Greece regarding the use of rapid tranquilisation and other coercive measures, and psychiatrists rely on their clinical experience for the management of agitation, or they follow guidelines from other countries. The application of such measures largely depends on local rules and practices [10], so it is expected that there may be wide variation in practice pattern within and between countries, as demonstrated in a recent European study [11]. We therefore believe that reports on chemical restraint are clinically relevant and studies on this topic should be regularly performed. The establishment of national guidelines in accordance with international proposed guidelines [12] may result in the harmonisation of the practices of Greek units with other units worldwide.

## Competing interests

The authors declare that they have no competing interests.

## Authors' contributions

NB conceived the study, and participated in its design and coordination and helped to draft the manuscript. GP participated in the study design and collected the data. VP helped in the data collection and the writing of the manuscript. All authors read and approved the final manuscript.
